# Predicting Early Awakening from Coma after Intracerebral Hemorrhage

**DOI:** 10.3389/fneur.2013.00162

**Published:** 2013-10-16

**Authors:** Diana Goodman, Scott E. Kasner, Soojin Park

**Affiliations:** ^1^Department of Neurology, Hospital of the University of Pennsylvania, Pennsylvania, PA, USA

**Keywords:** intracerebral hemorrhage, intracranial hemorrhage, ICH, prognosis, coma

## Abstract

**Introduction:** Given the high morbidity and mortality associated with intracerebral hemorrhage (ICH), family members, and healthcare providers base early supportive management decisions, at least in part, on expected prognosis. In the comatose patient with ICH, this short-term prognosis is most overtly characterized by regaining of consciousness.

**Design:** A retrospective consecutive cohort of 51 patients admitted to a neuroICU with ICH and admission Glasgow Coma Scale score ≤8 was identified. Logistic regression was performed to assess the association of baseline characteristics and treatment parameters associated with awakening.

**Results:** Awakening from coma was observed in 53% of ICH patients: 83% with an initial GCS score of 7–8, 43% with an initial score of 5–6, and 20% with an initial score of 3–4. Awakening from coma in the cohort of 27 patients who regained consciousness occurred in 59% of patients by day 2, 89% by day 7, and 96% by day 9. In multivariable analysis, only higher admission GCS score was associated with a greater likelihood of awakening from coma [OR 4.9 (95% CI 1.9–13) per two-point category, *p* = 0.001]. DNR status during the first 24 h was not associated with awakening but was at later time points.

**Conclusion:** GCS score is the predominant initial predictor of early awakening in patients who present in coma after ICH. Patients who regained consciousness typically did so within the first 9 days of hospital admission.

## Introduction

Intracerebral hemorrhage (ICH) carries the highest morbidity, mortality, and subsequent disability of all strokes (1–3). Given the current lack of effective treatments for ICH (2), family members and healthcare providers base supportive management decisions, at least in part, on expected prognosis. A number of clinical grading scales exist for predicting, from initial assessment, both in-hospital and 30 days mortality as well as functional outcome at hospital discharge, 1, 3, and 6 months after ICH (3–10). Intensive care units (and neurologic specialized units when available) tend to care for the most severe cases of ICH particularly when patients are comatose. In this acute setting, family members consistently ask when, or if, a patient will wake up (11). Beyond expected mortality and functional outcomes, what is often crucial for initial decision-making is short-term prognosis. In the comatose patient, this short-term prognosis is most overtly characterized by regaining of consciousness. Our study attempts to define the early natural history of comatose patients with ICH and to identify if and when comatose patients are likely to awaken after ICH.

## Materials and Methods

### Study population

After review and approval by the University of Pennsylvania Institutional Review Board in Philadelphia, Pennsylvania, we identified all patients with a diagnosis of ICH in our single tertiary hospital’s get with the Guidelines-Stroke database from 2006 through 2011. Additionally, we queried our neurological intensive care unit database for all patients with a primary diagnosis of spontaneous ICH with an initial GCS (Glasgow Coma Scale) of ≤8 (Table 1). All patients in the study were initially admitted to the neurologic ICU and treated in accord with AHA guidelines (1).

**Table 1:** **Flow chart of patient selection**.

Includes all stroke types^a^	2121 patients
ICH only	402 patients
ICH with altered consciousness	93 patients
ICH with initial GCS<8	51 patients

^a^ GWTG database 2006–2011.

^a^ ICU database 2010–2011 (36 total points >33 with spont ICH only >12 with GCS <8).

### Definition of coma

We chose to define coma as GCS ≤8 given the widespread clinical use of this score and our ability to track quantitative progression using daily GCS scores documented on nursing flow sheets. We identified 51 patients with ICH with an initial GCS score ≤8. Initial GCS score was defined as the lowest GCS score recorded in the first 24 h of hospital admission to account for the 30% of patients with ICH who decompensate in the first 24 h (2). Admission GCS score was categorized (3–4, 5–6, or 7–8) due to the modest sample size.

### Definition of aphasia

In the comatose population, aphasia was determined by an initial pre-comatose neurologic examination. In the absence of a pre-comatose history or examination, neuroimaging was evaluated to determine neuroanatomical probability of aphasia including lesions of the left temporal lobe.

### Data collected

Data obtained from medical records included age, sex, volume of ICH according to the ABC/2 rule (12), location of ICH, presence of IVH, admission BP, pre-existing cognitive impairment, prior stroke, antiplatelet use, and anticoagulant use. These factors were pre-selected as those that had previously been associated with ICH morbidity and mortality (3–10, 13). Presence of intubation on admission was also selected as a potential proxy for severity of coma. To account for any prominent treatment differences, documentation of EVD placement and/or surgical intervention was collected. Finally, we recorded code status (do not resuscitate, care de-escalation, and care withdrawal) throughout the duration of hospitalization, since withdrawal of care has been associated with poor long-term outcomes.

### Outcomes

Awakening from coma was defined as achieving a GCS motor score of 6 indicating the ability to follow commands, or 5 in patients with aphasia. This ability to interact with the external environment may be seen as proof of consciousness with greater reliability and validity than eye opening, and is typically seen prior to recovery of verbal responses, especially in intubated patients (14). Moreover, GCS component scores are documented multiple times per day in nursing flow sheets and medical records. After the first hospital day, the highest GCS score on each subsequent day was used to determine awakening. The secondary outcome variable was time to regaining of consciousness using the same definitions as above. In-hospital mortality, discharge disposition, and length of hospital stay were also collected.

### Statistical analysis

Univariate analysis employed *t*-tests or Wilcoxon ranked sum tests for continuous variables and chi-squared or Fisher’s exact tests for categorical data as appropriate. Variables associated with the outcome in univariate analysis at the *p* < 0.1 level were then tested in multivariable logistic regression. Analysis was performed using STATA SE 10.0. Final results were considered to be statistically significant at the *p* < 0.05 level. Given our available cohort size of 51 subjects and 27 who regained consciousness, we had power to identify 2–3 candidate variables associated with awakening.

## Results

We identified 51 ICH patients admitted with an initial GCS score ≤8. The median length of stay in the hospital was 10 (IQR 4–24) days. 53% (27 of 51) experienced in-hospital awakening and 47% (24 of 51) remained comatose at time of death or hospital discharge. Table 2 summarizes the baseline characteristics of both groups. In univariate analysis, subjects were more likely to awaken if they presented with higher admission GCS, smaller ICH volume, and less IVH. Interventions after admission were similar in both groups.

**Table 2:** **Baseline characteristics for each group**.

	Pts with in-hospital Awakening [*n* = 27, *N* (%)]	Pts remaining Comatose [*n* = 24, *N* (%)]	*p* Value
Age, years (mean ± SD, median)	59 ± 16, 61	64 ± 18, 58	0.30
Sex, % female	14 (52)	10 (42)	0.47
Admission GCS			<0.001
3–4	4 (15)	16 (66)	
5–6	3 (11)	4 (17)	
7–8	20 (74)	4 (17)	
Pre-ICH cognitive impairment	1 (4)	4 (17)	0.18
Hx of stroke	3 (11)	5 (21)	0.45
Hx of ICH	0	3 (12)	0.10
Intubated on arrival	17 (63)	20 (83)	0.13
Pre-ICH anticoagulation	4 (15)	6 (25)	0.49
Admission BP
SBP mean (±SD)	188 ± 31	196 ± 38	0.40
DBP mean (±SD)	109 ± 25	108 ± 25	0.73
Aphasia on admission	4 (15)^c^	4 (17)^d^	1.0
ICH Location^a^			0.87
Lobar	6 (22)	8 (33)	
Deep	19 (70)	16 (67)	
Infratentorial	1 (4)	0	
ICH volume, cm^3^^b^			0.03
<30	16 (59)	9 (38)	
30–60	9 (33)	6 (25)	
>60	1 (4)	8 (33)	
IVH	17 (63)	21 (88)	0.024
Surgery	4 (15)	4 (17)	1.0
EVD placed	7 (26)	5 (21)	0.75
Intraventricular TPA	1 (4)	0	1.0
DNR in first 24 h^e^	1 (4)	4 (17)	0.18
DNR after 24 h^e^	4 (15)	13 (54)	0.006

^a^ 1 patient with IVH only.

^b^ 1 patients with missing scan.

^c^ 3 patients diagnosed clinically, 1 radiographically.

^d^ 1 patient diagnosed clinically, 3 radiographically.

^e^ Including de-escalation and withdrawal of care.

Early DNR and care limitation orders in the first 24 h were used more frequently in patients who ultimately remained comatose (17 vs. 4%), but the difference was not statistically significant (Table 2). Patients remaining comatose beyond 24 h had their code status changed significantly more often than those who awoke (54 vs. 15%). For all patients who had care withdrawn or de-escalated, median day of care withdrawal or de-escalation was 3.5 days.

**Figure 1:**
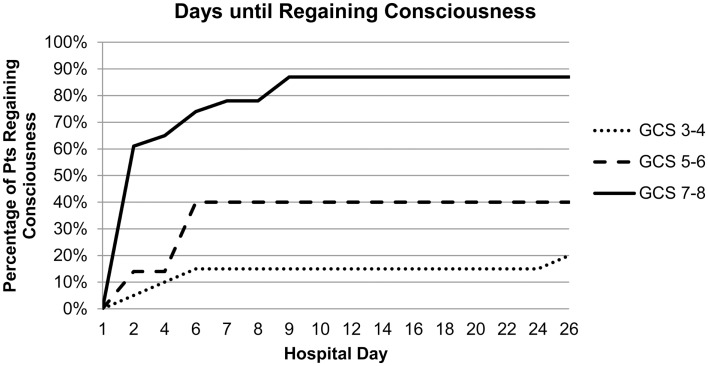
**Admission GCS category and number of hospital days until awakening from coma**.

Admission GCS category was strongly associated with awakening and time to awakening from coma (Figure 1). In multivariable analysis, higher admission GCS score was associated with a higher likelihood of awakening from coma [OR 4.9 (95%CI 1.9–13) per category, *p* = 0.001], while ICH volume and IVH were no longer associated with awakening (Table 3). This model had excellent ability to discriminate which patients would awaken in-hospital (*C*-statistic 0.89).

**Table 3:** **Multivariable analysis: association of prognostic factors with awakening from coma**.

	Odds ratio (95% CI)	*p* Value
Admission GCS per category	4.88 (1.90–12.49)	0.001
Volume ICH	0.98 (0.95–1.01)	0.12
IVH	0.47 (0.07–3.32)	0.45

Goodness-of-fit (Hosmer–Lemeshow) test *p* = 0.77; *C*-statistic 0.89.

A sensitivity analysis was performed using motor score of 6 as the primary outcome (rather than allowing for 5 in aphasic subjects) for all patients without a substantial change in these results.

Awakening from coma in the cohort of 27 patients who regained consciousness occurred in 59% of patients by day 2, 89% by day 7, and 96% by day 9. The last patient did not achieve a motor score of 6 until hospital day 26. Median length of stay for patients who awoke was similar to those who survived and remained comatose [20 (IQR 10–33) vs. 17 (IQR 9–40)]. Tracheostomy (trach) and percutaneous endoscopic gastrostomy (PEG) tube placement was performed in 12 patients who awoke with another 5 patients requiring PEG tube placement only. In contrast, trach and PEG tube placement was performed in only four patients who remained comatose. All of the patients who awoke survived the initial hospitalization and the majority were discharged to home or rehabilitation facilities (67%, 18/27) with four discharged to a skilled nursing facility, four to hospice, and one to a ventilator weaning facility. In contrast, 71% (17/24) of those who remained comatose died, four were discharged to hospice, two to a skilled nursing facility, and six to a ventilator weaning facility.

## Discussion

The time to awakening, not previously described in the literature to our knowledge, was typically early. Nearly all who awoke did so by days 7–9. The one patient who awoke later did not consistently remain conscious and was discharged to a skilled nursing facility. Establishing the natural history of awakening from coma in patients with ICH is particularly useful when counseling families about trach and PEG tube placement. Per policy, these procedures are performed within 14 days of intubation. In our observational study, few of these procedures were performed in comatose patients. The decision to proceed with trach and PEG placement in comatose patients is a manifestation of continued hope of awakening. If confirmed with further studies, regaining consciousness by day 9 would be a reasonable goal for patients and their families considering trach and PEG placement.

While ultimate functional outcome was not explored in this study, the majority of patients who awoke were discharged to home or rehabilitation facilities. The majority of patients who remained comatose died during hospitalization or were discharged to hospice. Furthermore, the four patients who awoke but were discharged to hospice had subsequently lost consciousness during hospitalization. Three of those four patients developed secondary infections during their hospital stay. Awakening is therefore a necessary but not sufficient indicator of ultimate discharge disposition.

We found that the GCS score during the first 24 h is the predominant initial predictor of awakening from coma after ICH. About 83% of patients with an initial GCS score of 7–8, 43% with an initial GCS score of 5–6, and 20% with an initial GCS score of 3–4 awoke. While large ICH volume and presence of IVH showed a trend toward association with remaining comatose, in the end only GCS score emerged as an independent predictor of awakening.

Our study has several potential limitations. All patients in the study were initially admitted to the neurologic ICU of a single academic medical center and thus our results may not be generalizable to other settings. The sample size is modest, limiting our power for analysis of additional factors and interactions. While specific details regarding potential differences in the care of individual patients were not fully accounted for, we did ascertain that patients received similar interventions in each group and medical treatment was in accord with AHA guidelines (1). Our data are subject to a withdrawal of care bias. This is regrettably unavoidable in the severely injured ICH population. Patients who had care withdrawn or de-escalated remained comatose; but undoubtedly those who remained comatose were more likely to have care withdrawn. We distinguished code status and care limitations in the first 24 h from those beyond 24 h in an attempt to account for this bias. We found no statistical difference in code status and care limitations between groups in the first 24 h. A larger prospective trial excluding patients with care withdrawn would be needed to further address this bias.

In summary, our findings suggest patients who regained consciousness did so within the first 7–9 days of hospital admission. Establishing the natural history of awakening will be extremely helpful in counseling families about continued aggressive care including trach and PEG placement which are typically performed by day 14. Notably, early GCS score is the predominant predictor of early awakening from coma after ICH. A larger prospective study looking both at time to awakening and prognostic factors of awakening is needed to develop a clinically useful tool for counseling families about both the short and long-term prognosis after ICH.

## Conflict of Interest Statement

The authors declare that the research was conducted in the absence of any commercial or financial relationships that could be construed as a potential conflict of interest.
